# Enzalutamide in metastatic hormone-sensitive prostate cancer: results from the international, multicentre, real-world ARON-3 study

**DOI:** 10.1038/s41391-025-01067-3

**Published:** 2026-01-08

**Authors:** Thomas Büttner, Mimma Rizzo, Laura Bernal Vaca, Javier Molina-Cerrillo, Teresa Alonso-Gordoa, Alvaro Juárez, Tomas Buchler, Emmanuel Seront, Jakub Kucharz, María Natalia Gandur Quiroga, Maria T. Bourlon, Martin Bögemann, Jindrich Kopecky, Pasquale Rescigno, Ondrej Fiala, Alejo Rodriguez-Vida, Zin W. Myint, Yüksel Ürün, Sarah Scagliarini, Alexandr Poprach, Diana Matthews, Haoran Li, Alessandro Rizzo, Alessandra Mosca, Edoardo Lenci, Bohuslav Melichar, Mike Wenzel, Philipp Mandell, Francesco Massari, Emmanuel S. Antonarakis, Matteo Santoni

**Affiliations:** 1 Department of Urology and Paediatric Urology, University Hospital Bonn, Bonn, Germany; 2 Division of Medical Oncology, Azienda Ospedaliera Universitaria Consorziale - Policlinico di Bari, Bari, Italy; 3 Servicio de Oncología, Clínica Universitaria Colombia – Clínica de Marly, Bogotá, Colombia; 4 Medical Oncology Department, Hospital Universitario Ramón y Cajal, Madrid, Spain; 5 Department of Urology, Jerez University Hospital, Jerez, Spain; 6 Department of Oncology, Second Faculty of Medicine, Charles University and University Hospital Motol, Prague, Czech Republic; 7 Department of Medical Oncology, Cliniques Universitaires Saint Luc, Brussel, Belgium; 8 Department of Uro-Oncology, Maria Sklodowska-Curie National Research Institute of Oncology, Warsaw, Poland; 9 Division of Genitourinary Medical Oncology, Instituto de Oncologia Ángel H. Roffo, Buenos Aires, Argentina; 10 Hematology and Oncology Department, Instituto Nacional de Ciencias Médicas y Nutrición Salvador Zubirán, Mexico City, Mexico; 11 Department of Urology, University Hospital Münster, Münster, Germany; 12 West German Cancer Center, Münster, Germany; 13 Department of Clinical Oncology and Radiotherapy, University Hospital Hradec Kralove, Hradec Kralove, Czech Republic; 14 Translational and Clinical Research Institute, Centre for Cancer, Newcastle University, Newcastle upon Tyne, UK; 15 Department of Oncology and Radiotherapeutics, Faculty of Medicine, University Hospital in Pilsen, Charles University, Pilsen, Czech Republic; 16 Biomedical Center, Faculty of Medicine in Pilsen, Charles University, Pilsen, Czech Republic; 17 Medical Oncology Department, Hospital del Mar, Barcelona, Spain; 18 Division of Medical Oncology, Markey Cancer Center, University of Kentucky, Lexington, KY USA; 19 Medical Oncology, Ankara University Medical Faculty, Ankara, Türkiye; 20 Department of Medical Oncology, AORN “A. Cardarelli”, Naples, Italy; 21 Masaryk Memorial Cancer Institute, Brno, Czech Republic; 22 Faculty of Medicine, Masaryk University, Brno, Czech Republic; 23 Velindre Cancer Centre, Velindre Road, Whitchurch, Cardiff, Wales; 24 Division of Medical Oncology, Department of Internal Medicine, University of Kansas Cancer Center, Westwood, KS USA; 25 S.S.D. C.O.r.O. Bed Management Presa in Carico, TDM, IRCCS Istituto Tumori “Giovanni Paolo II”, Bari, Italy; 26 Oncology Department, Candiolo Cancer Institute, IRCCS-FPO, Torino, Italy; 27 Medical Oncology Unit, Azienda Ospedaliera Ospedali Riuniti Marche Nord, Pesaro, Italy; 28 Department of Oncology, Faculty of Medicine and Dentistry, Palacký University, Olomouc, Czech Republic; 29 Department of Urology, Goethe University Frankfurt, University Hospital, Frankfurt am Main, Germany; 30 Martini-Klinik Prostate Cancer Center, University Hospital Hamburg-Eppendorf, Hamburg, Germany; 31 Medical Oncology, IRCCS Azienda Ospedaliero-Universitaria di Bologna, Bologna, Italy; 32 Department of Medical and Surgical Sciences (DIMEC), University of Bologna, Bologna, Italy; 33 University of Minnesota, Masonic Cancer Center, Minneapolis, MN USA; 34 Medical Oncology Unit, Macerata Hospital, Macerata, Italy

**Keywords:** Prostate cancer, Outcomes research

## Abstract

**Background:**

Enzalutamide (ENZA), a next-generation non-steroidal androgen receptor (AR) inhibitor, plays a pivotal role in the management of both hormone-sensitive (HSPC) and androgen deprivation-resistant prostate cancer (ARPC). This paper presents real-world clinical outcomes of ENZA in a subgroup of metastatic HSPC (mHSPC) patients included in the ARON-3 study.

**Methods:**

Clinical information was extracted retrospectively from medical records at 29 cancer centres in 9 countries worldwide. Overall Survival (OS) was calculated from starting ENZA to death from any cause and the time on treatment (ToT) from ENZA initiation to discontinuation for any reason. The Kaplan–Meier method was used to estimate OS and ToT. PSA90 was defined as a ≥90% PSA reduction from baseline, and PSA0.2 as the achievement of an ultra-low PSA level ≤0.2 ng/ml. Adverse events (AEs) were categorised according to Common Terminology Criteria for Adverse Events v5.0.

**Results:**

The study population comprised 424 patients treated with ENZA for mHSPC, of whom 80 (19%) had lymph node-only metastases, 265 (63%) bone-only metastases, and 50 (12%) visceral metastases. 273 patients (64%) had synchronous metastases and 151 (36%) had developed metachronous metastases. A total of 228 patients were diagnosed with low-volume disease, and 196 patients (46%) with high-volume disease. The median ToT was 31.8 months, and the median OS was not reached. The median time to PSA90 (achieved in 76% of patients) and PSA0.2 (59% of patients) was 6.0 months and 8.3 months, respectively. Statistically significant associations were identified between lymph node-only patterns, PSA90 and ultra-low PSA responses, and longer treatment duration and better overall survival. Grade 3–4 AEs were observed in 9% of patients <70 years and in 10% ≥70 years.

**Conclusions:**

Real-world clinical practice corroborates the findings from clinical trials, confirming the effectiveness and safety of ENZA in mHSPC patients.

## Introduction

The treatment landscape for metastatic castration-sensitive prostate cancer (mHSPC) has been substantially transformed by the introduction of next-generation androgen receptor pathway inhibitors (ARPIs) [1]. Enzalutamide (ENZA) is a next-generation non-steroidal androgen receptor (AR) inhibitor that competitively binds to the AR ligand-binding domain with higher affinity than first-generation antiandrogens and blocks androgen binding to the AR, nuclear translocation and DNA binding of liganded AR and coactivator recruitment, thereby consistently reducing proliferation and inducing cell death of prostate cancer cells [2]. The pharmacokinetic profile of ENZA is characterised by stable plasma concentrations and low intersubject variability [3]. These properties have enabled ENZA to establish a leading role in the treatment of both non-metastatic and metastatic castration-resistant prostate cancer (mARPC) [4, 5]. More recently, it was the first ARPI to demonstrate an overall survival benefit in both non-metastatic [6] and metastatic HSPC (mHSPC) [7, 8].

The international, double-blind, phase III ARCHES trial randomised 1150 patients with mHSPC to ENZA plus androgen deprivation therapy (ADT) or placebo plus ADT [7]. At a median follow-up of 44.6 months, ENZA plus ADT reduced the risk of death by 34% compared to placebo plus ADT [8]. Furthermore, the risk of disease progression was reduced by 37% with a median extension of radiographic progression-free survival (rPFS) of ~11 months at the first pre-planned analysis [7]. The clinical benefit of enzalutamide plus ADT was independent of prior local treatment, disease volume, and risk classification [9]. Furthermore, ENZA plus ADT demonstrated an advantage in time to prostate-specific antigen (PSA) progression, time to subsequent antineoplastic therapy, time to first symptomatic skeletal event, and time to castration resistance in all patient subgroups [10].

The efficacy results, combined with the drug manageability [3, 11], have encouraged the widespread use of ENZA in the real-world mHSPC clinical practice. In the absence of clinical trials comparing guideline-recommended ARPIs, and with the advent of taxane-containing triple therapy in mHSPC [12, 13], real-world data represent a valuable source for identifying the clinical and biological subgroups most likely to benefit from a particular treatment option and to optimise the sequencing of therapy.

ARON-3 (ClinicalTrials.gov identifier, NCT06200558) is a retrospective, multicentre, international study that collected real-world data from prostate cancer patients. Real-world experience with ENZA in the mHSPC population is the focus of this analysis.

## Methods

### Study design and population

Adult patients who received ENZA between 1 January 2020 and 31 May 2024 for a diagnosis of metastatic castration-sensitive prostate cancer were selected from the ARON-3 database and included in this study. Clinical and pathological information was extracted from the patients’ medical and pathological reports at 29 oncology centres in 9 countries worldwide. Data on age, tumour histology, Eastern Cooperative Oncology Group Performance Status (ECOG-PS), number and location of metastatic sites, previous definitive surgery or radiotherapy, enzalutamide dose and duration, and PSA response during ENZA treatment were available. Patients with missing clinical or outcome data were excluded from further analysis.

The protocol was approved on 18 April 2024 by the Ethics Committee of the coordinating centre (Marche Region - Italy - No. 2024 20, ‘ARON-3 Study’ protocol) and the Institutional Review Boards of each participating site. The study was conducted in accordance with the Declaration of Helsinki on human experimentation and Good Clinical Practice standards.

### Study objectives

The primary objective of the analysis was to evaluate real-world clinical outcomes in ENZA-treated mHSPC patients.

We recorded time on treatment duration (ToT) and overall survival (OS). Time on treatment (ToT) was defined as the time from ENZA initiation to discontinuation for any reason, including toxicity. OS was calculated from starting ENZA to death from any cause.

PSA90 was defined as a decrease in PSA level ≥90% from baseline, while PSA0.2 was defined as the achievement of an ultra-low PSA level ≤0.2 ng/mL, as previously described [14].

Severe adverse events (AEs), defined as grade ≥3 according to the Common Terminology Criteria for Adverse Events (CTCAE) v5.0 or leading to dose reduction or treatment interruption were included in the data collection.

### Statistical considerations

OS was estimated by the Kaplan–Meier method and compared between subgroups using the log-rank test. The Kaplan–Meier method was also used to calculate median follow-up time and ToT. Cox proportional hazards models were used to compare multivariable effects on patient survival and calculate hazard ratios (HRs) and 95% confidence intervals (CIs). For comparisons of categorical variables between groups, the chi-square test and Fisher’s exact test were applied. *P* values of <0.05 were considered to be statistically significant. Statistical analyses were performed using MedCalc version 19.6.4 (MedCalc Software, Broekstraat 52, 9030 Mariakerke, Belgium).

## Results

### Patient population

A total of 424 patients treated with ENZA for mHSPC were identified in the ARON-3 dataset (see Supplementary Fig. S1). The median follow-up time was 18.7 months (95% CI 16.9–50.0). At the time of this analysis, 53 patients (13%) had died.

The median age was 72 years (range: 42–91 years). ECOG-PS was 0 in 233 patients (55%). At initial diagnosis, grade group was 4–5 in 271 patients (63%). 273 patients (64%) had de novo (synchronous) metastatic disease, while 151 (36%) had developed metachronous metastases. Eighty patients (19%) had lymph node metastases only (M1a), and 294 (69%) had bone metastases (M1b). Visceral metastases were observed in 50 patients (12%), including 47 (11%) patients with lung metastases, 11 (3%) liver metastases and 1 (<1%) brain metastases. Low-volume disease per CHAARTED criteria [15] was reported in 228 patients (54%). The median PSA level at baseline was 18.3 ng/mL (range 0.1–9943 ng/mL). In 71 patients (17%), enzalutamide was administered after docetaxel. Patient characteristics are presented in Table 1.

**Table 1:** Patients’ baseline demographic and clinical characteristics.

Patients	Overall 424 (%)
Age, years (y)	72
Range	42–91
ECOG-PS	
0	233 (55)
1	159 (38)
≥2	32 (7)
Grade Group at initial diagnosis	
1	15 (4)
2–3	138 (33)
4–5	271 (63)
Metastatic stage	
de novo	273 (64)
metachronous	151 (36)
Disease volume	
Low-volume	228 (54)
High-volume	196 (46)
Metastatic stage	
Exclusive metastases to distant lymph nodes (M1a)	80 (19)
Bone metastases (M1b)	294 (69)
Visceral metastases (M1c)	50 (12)
Visceral metastases	50 (12)
Lung metastases	47 (11)
Liver metastases	11 (3)
Previous treatment with docetaxel for mHSPC	71 (17)
Radiotherapy for localised HSPC	47 (11)
Radical prostatectomy for localised HSPC	104 (25)
PSA value at enzalutamide initiation	
Median (ng/ml)	18.3
Range (ng/ml)	0.1–9943.0

Ninety of the 126 patients (71%) progressed during ENZA therapy received further treatments for metastatic androgen deprivation-resistant prostate cancer (mARPC): 46 patients received docetaxel, 12 cabazitaxel, 9 radium-223, 10 abiraterone acetate, 5 lutetium-177-PSMA-617, 6 olaparib, and 4 were enrolled into experimental clinical trials.

### Survival analysis

**Fig. 1:**
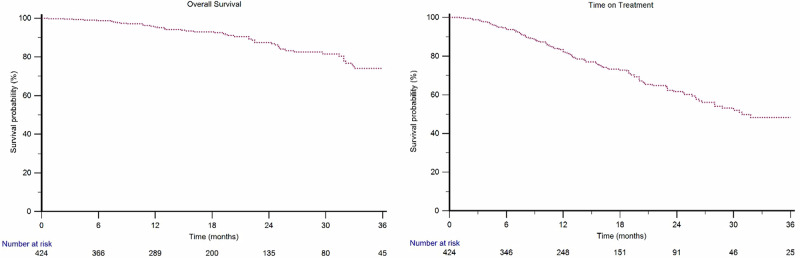
Overall survival and time on treatment in mHSPC patients treated with enzalutamide.

The median OS in the overall study population was not reached (NR, Fig. 1) after a median follow-up time of 18.7 months (95% CI 16.9–50.0).

**Fig. 2:**
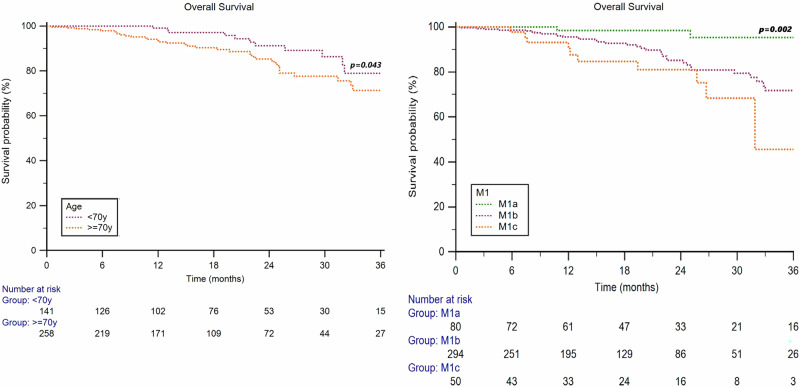
Overall survival from the start of enzalutamide treatment to death for any cause in mHSPC patients stratified by selected clinical characteristics (age, metastatic stage).

The median OS was NR in patients with ECOG-PS = 0, 1 and 2, with 2-year-OS rates of 93%, 83% and 80%, respectively (*p* = 0.025). Stratified by age, the median OS was longer in patients aged <70 years (NR vs 44.4 months, 95% CI 42.7–44.8, HR 0.50, 95% CI 0.28–0.88, *p* = 0.016, Fig. 2), with a 2-year-OS rate of 91% vs 80% (*p* = 0.043).

The median OS was NR in both high- and low-volume patients (*p* = 0.118), with a 2-year-OS rate of 85% and 89%, respectively (*p* = 0.411). The median OS was NR in M1a, M1b and M1c patients (*p* = 0.002, Fig. 2; M1b vs M1a: HR 3.43, 95% CI 1.81–6.53; M1c vs M1a: HR 6.55, 95% CI 2.38–18.04; M1c vs M1b: HR 1.91, 95% CI 0.77–4.75), with a 2y-OS rate of 98% vs 88% vs 80% (*p* = 0.003).

We further stratified patients basing on the type of visceral metastases, showing that the median OS was decreased in both patients with lung (HR 2.81, 95% CI 1.13–6.99, *p* = 0.026, Supplementary Fig. S3) or liver metastases (HR 13.54, 95% CI 1.57–116.54, *p* = 0.018, Supplementary Fig. S3).

In the 71 patients treated with docetaxel before ENZA, the median OS was NR, with a 2-year-OS rate of 76% (HR 1.62, 95% CI 0.79–3.30, *p* = 0.181).

### Time on treatment

The median ToT with ENZA was 31.8 months (95% CI 26.6–45.0, Fig. 1). The median ToT was NR in patients with ECOG-PS = 0, 26.0 months (95% CI 19.1–45.0) in subjects with ECOG-PS = 1 and 19.5 months (95% CI 10.3–43.9) in the ECOG-PS = 2 subgroup (*p* < 0.001, Supplementary Fig. S2). No significant differences were found between patients aged <70 years vs ≥70 years (30.9 months, 95% CI 23.0–45.0, vs 36.1 months, 95% CI 24.8–43.9, *p* = 0.853).

The median ToT was longer in patients with low-volume disease (43.3 months, 95% CI 30.6–45.0, vs 28.0 months, 95% CI 20.6–31.8, *p* = 0.007, Supplementary Fig. S2).

The median ToT was NR in M1a patients and 28.8 months (95% CI 23.0–43.3) in those with M1b/M1c disease (*p* = 0.004, Supplementary Fig. S2). The median ToT was significantly shorter in M1b patients compared to subjects with other metastatic sites (28.0 months, 95% CI 21.4–36.1, vs 43.3 months, 95% CI 28.0–45.0, *p* = 0.045, Supplementary Fig. S2). No significant differences were found between patients with or without visceral metastases (30.0 months, 95% CI 23.7–31.3, vs 31.8 months, 95% CI 26.0–45.0, *p* = 0.912). We further stratified patients basing on the type of visceral metastases, showing no significant differences between patients with and without lung (HR 0.99, 95% CI 0.57–1.72) or liver metastases (HR 1.52, 95% CI 0.39–5.89).

The median ToT was significantly shorter in patients treated with previous docetaxel (23.0 months, 95% CI 14.3–31.8, vs 36.1 months, 95% CI 26.6–45.0, HR 1.78, 95% CI 1.11–2.85, *p* = 0.016, Supplementary Fig. S2).

### PSA dynamics

**Fig. 3:**
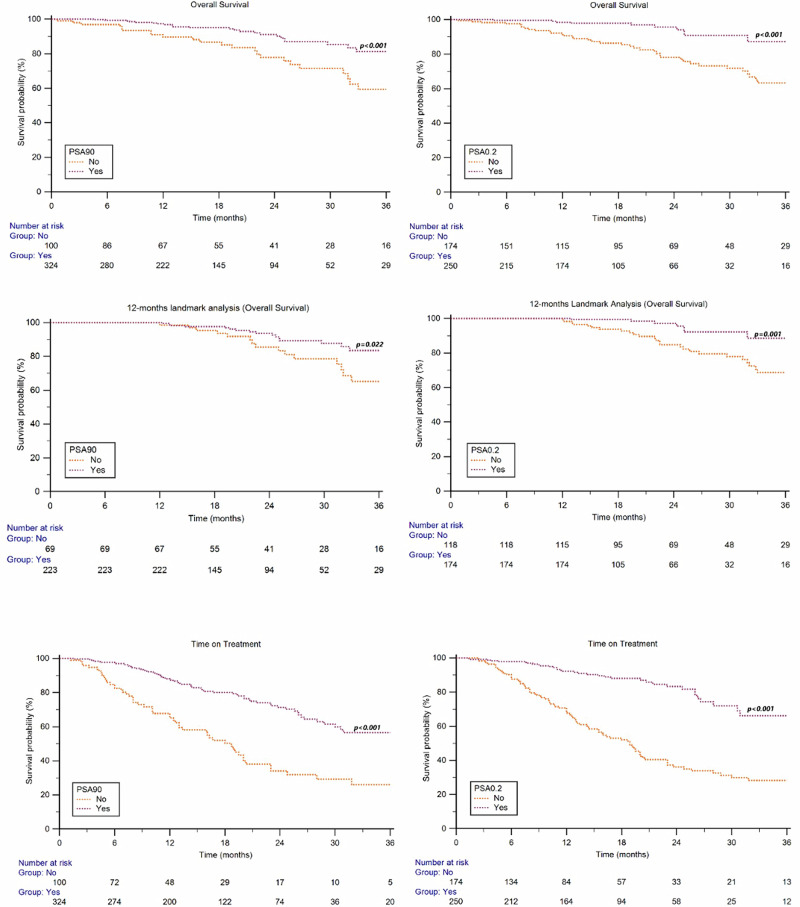
Overall Survival, 12-month landmark survival, and time on treatment in mHSPC patients treated with enzalutamide who achieved PSA90 and PSA0.2 responses.

PSA90 response was observed in 324 patients (76%). The median time to PSA90 was 6.0 months (95% CI 5.3–23.9); 109 patients (26%) achieved PSA90 within 3 months and 215 patients (51%) after 3 months. The median OS was significantly longer in patients who achieved PSA90 compared to those who did not (NR vs 42.7 months, 95% CI 32.1–43.9, HR 0.36, 95% CI 0.20–0.65, *p* < 0.001, Fig. 3). The 2y-OS rate was 91% in the PSA90 subgroup, contrasting with 78% for those who did not achieve PSA90 response (*p* = 0.018), and it was 93% in patients who achieved PSA90 within 3 months and 83% in subjects who achieved PSA90 later (*p* = 0.048). PSA increases were observed in 27 patients (6%), with a median time to PSA progression of 5.6 months (95% CI 3.7–13.4).

In the 12-month OS landmark analysis, the median OS was NR in both patients who achieved or not PSA90 response (HR 0.44, 95% CI 0.22–0.89, *p* = 0.022, Fig. 3), with a 2y-OS rate of 94% in the PSA90 subgroup and 86% in those who did not achieve PSA90 response (*p* = 0.097).

PSA0.2 was observed in 250 patients (59%). The median time to PSA0.2 was 8.3 months (95% CI 6.9–9.8); 94 patients (22%) obtained PSA0.2 within 3 months, 156 patients (37%) after 3 months. The median OS was significantly longer in patients who achieved PSA0.2 (NR vs 44.4 months, 95% CI 40.9–44.8, HR 0.29, 95% CI 0.17–0.51, *p* < 0.001, Fig. 3). The 2y-OS rate was 96% in the PSA0.2 subgroup and 78% in patients who did not achieve a PSA0.2 response (*p* < 0.001), being 98% and 86% in patients who achieved PSA0.2 within or after 3 months (*p* = 0.003).

In the 12-month OS landmark analysis, the median OS was NR in both patients who achieved or not PSA0.2 response (HR 0.34, 95% CI 0.18–0.65, *p* = 0.001, Fig. 3), with 2-year-OS rates of 97% and 85% in the PSA0.2 and non-PSA0.2 subgroups (*p* = 0.005).

With respect to ToT, it was significantly longer in both patients who achieved PSA90 (NR vs 18.7 months, 95% CI 13.0–45.0, HR 0.24, 95% CI 0.16–0.37, *p* < 0.001, Fig. 3) and PSA0.2 (NR vs 18.9 months, 95% CI 14.4–20.6, HR 0.22, 95% CI 0.15–0.32, *p* < 0.001, Fig. 3).

### Safety

Grade 3–4 AEs were reported in 9% of the study population. The most common severe AEs were fatigue (9%) and hypertension (2%). In patients aged ≥70 y, the incidence of severe AEs was 10%, with G3-G4 fatigue reported in 10% of elderly patients (Supplementary Table S1). Full-dose recipients were 93%, while 7% received initial reduced dose; 42 patients (9%) reduced ENZA dose due to severe AEs.

### Univariable and multivariable analyses

In the overall study population, age, ECOG-PS, PSA90 and PSA0.2 were significantly associated with OS at univariable analysis (Table 2). At multivariable analysis, only age and PSA0.2 were significantly correlated with OS (Table 2).

**Table 2:** Univariable and multivariable analyses.

Overall survival	Univariable Cox regression		Multivariable Cox regression	
	HR (95% CI)	*p*-value	HR (95% CI)	*p*-value
Age (≥70 years vs <70 years)	2.23 (1.14−4.36)	**0.019**	2.33 (1.19–4.57)	**0.014**
ECOG-PS (≥2 vs <2)	1.57 (1.04−2.37)	**0.032**	1.32 (0.82−2.11)	0.249
Grade Group 1 vs Grade Group ≥2	0.98 (0.62−1.58)	0.956		
De novo vs metachronous	0.91 (0.52−1.59)	0.729		
High volume vs low volume	1.53 (0.89−2.64)	0.121		
Previous docetaxel (yes vs no)	1.53 (0.82−2.86)	0.185		
PSA90 (yes vs no)	0.41 (0.24−070)	**0.001**	0.81 (0.43−1.51)	0.505
PSA0.2 (yes vs no)	0.25 (0.13−0.49)	**<0.001**	0.27 (0.12−0.57)	**<0.001**
ENZA dose reductions (yes vs no)	1.34 (0.61−3.88)	0.579		

Bold values indicate statistical significance *p* < 0.05.

## Discussion

To the best of our knowledge, this subgroup of the ARON-3 study represents the largest international real-world dataset of patients receiving ENZA for mHSPC published to date. Present results confirm the favourable survival rates associated with this treatment in the castration-sensitive disease setting. OS data strongly resemble those of ARCHES, where 2-year survival rates were at 86% and median OS was not reached in a median follow-up of 44.6 months [8]. The considerably short median follow-up of 18.7 months in the present cohort represents one of the main limitations, but is expected as a new drug is introduced into clinical practice many years after starting enrolment in a clinical trial.

Examining our patients’ characteristics, they presented with a similar median age (70 vs. 72 years), rates of de novo mHSPC (64 vs. 63%), Grade Group 4-5 (63 vs. 67%), presence of visceral metastases (11 vs. 12%) and pre-treatment with docetaxel (17 vs. 18%) to the ARCHES. In contrast, the ARON-3 patients had higher median PSA levels (18.3 vs. 5.1 ng/mL), and the present dataset had more patients presenting with bone-only metastases (63 vs. 47%), low volume of diseases (54 vs. 38%) and ECOG PS > 0 (45 vs. 22%) [8]. Despite these differences, our data validate the survival results of the phase III trial in a global real-world setting, helping to overcome eventual selection bias. Notably, mHSPC patients with high ECOG PS scores, for whom the mortality risk is significantly higher [16], are generally excluded from clinical trials. In the present study, the efficacy and toxicity outcomes of enzalutamide were confirmed for 45% of patients with ECOG PS > 0, including 7% with ECOG PS 2 (an exclusion criterion for ARCHES trial).

ToT further clinically expands PFS, by considering patients that had to abort treatment [17]. In the ARON-3 cohort, we found benefits in ToT especially for patients with an ECOG PS = 0 and a lower metastatic burden, marked by low-volume disease according to CHAARTED criteria, lymph-node metastases only or an absence of bone metastases. While these criteria represent known prognostic factors in mHSPC [18], they may also aid in patient selection. “Triple therapy” schemes in mHSPC, combining taxane chemotherapy and ARPI, have demonstrated benefits over ADT alone, especially in men with high-volume disease and beyond M1a stage [13]. These ARON-3 data further add to the medical need for treatment intensification in these patients.

Our data also provide useful insights into the real-world safety profile of ENZA in mHSPC. Notably, with a 9% rate of severe AEs, predominantly due to the onset of fatigue, ENZA exhibited an excellent safety profile. Furthermore, elderly patients >70 years were hardly more affected by these. When interpreting safety data from real-world analyses, the lack of standardised AE measures and follow-up protocols has to be considered and could have resulted in under-reporting of adverse events. However, the safety profile of ENZA was similarly favourable in ARCHES, with a comparably low occurrence of the AEs of special interest hypertension, rash or skeletal events [7]. Among the ARPIs currently available for mHSPC, these observations may therefore be interpreted as an argument in favour of ENZA in patients who do not tolerate pronounced toxicity and skin reactions. Comparative data on this topic should be sought.

In regard of factors associated with survival benefits, present results highlight the role of PSA response patterns, PSA90 and especially PSA0.2, as valuable on-treatment predictors of both OS and ToT. A post-hoc analysis of the ARCHES study revealed that patients with undetectable (<0.2 ng/mL) PSA experienced improved rPFS and OS versus patients with detectable (≥0.2 ng/mL) PSA levels [19]. Achieving both PSA90 and PSA0.2 was associated with improved survival in mHSPC patients treated with the alternative ARPIs regimens, apalutamide, darolutamide + docetaxel or abiraterone acetate + prednisolone [14, 20–22]. Our findings align with existing data and add the missing evidence for ENZA in mHSPC. We confirm the prognostic significance of PSA90 and PSA0.2 in a large real-world cohort of ENZA-treated mHSPC patients. Median time to PSA90 and PSA0.2 of respective 6.0 months and 8.3 months, indicate that the velocity of PSA response to ENZA treatment has to be expected at moderate speed, as reported in an American real-world mHSPC cohorts by Lowentritt et al. [23]. With this specificity in mind, PSA90 and PSA0.2 provide clinicians with easy-to-use and cost-effective prognostic markers that are highly useful in ENZA treatment.

In addition to the above-outlined and discussed limitations, our study should be interpreted in its light of the non-randomised, retrospective design with mid-term follow-up duration. Moreover, some missing data, as well as other not reported variables may have influenced cancer-control outcomes, such as the used staging modality for metastases (conventional vs. molecular imaging) and patients’ comorbilities. Therefore, a potential selection bias cannot be completely ruled out. In conclusion, the present data from ARON-3 confirmed the favourable prognosis and safety profile of ENZA treatment in mHSPC in an international real-world setting. Safety was consistent with the published literature even in patients aged >70 years. Lower metastatic burden with lymph node-only patterns, and achieving ultra-low PSA responses, were associated with prolonged time on treatment and improved overall survival.

## Supplementary information

Supplementary Materials

## Data Availability

The datasets generated during and/or analysed during the current study are available from the corresponding author on reasonable request.

## References

[1] Kwon WA, Song YS, Lee MK. Strategic advances in combination therapy for metastatic castration-sensitive prostate cancer: current insights and future perspectives. Cancers. 2024;16:3187. 10.3390/cancers16183187. 39335158 10.3390/cancers16183187 PMC11430187

[2] Tran C, Ouk S, Clegg NJ, Chen Y, Watson PA, Arora V, et al. Development of a second-generation antiandrogen for treatment of advanced prostate cancer. Science. 2009;324:787–90. 19359544 10.1126/science.1168175 PMC2981508

[3] Gibbons JA, Ouatas T, Krauwinkel W, Ohtsu Y, van der Walt JS, Beddo V, et al. Clinical pharmacokinetic studies of enzalutamide. Clin Pharmacokinet. 2015;54:1043–55. 10.1007/s40262-015-0271-5. 25917876 10.1007/s40262-015-0271-5 PMC4580721

[4] Hussain M, Fizazi K, Saad F, Rathenborg P, Shore N, Ferreira U, et al. Enzalutamide in men with nonmetastatic, castration-resistant prostate cancer. N Engl J Med. 2018;378:2465–74. 10.1056/NEJMoa1800536. 29949494 10.1056/NEJMoa1800536 PMC8288034

[5] Beer TM, Armstrong AJ, Rathkopf D, Loriot Y, Sternberg CN, Higano CS, et al. Enzalutamide in men with chemotherapy-naïve metastatic castration-resistant prostate cancer: extended analysis of the phase 3 PREVAIL study. Eur Urol. 2017;71:151–4. 10.1016/j.eururo.2016.07.032. 27477525 10.1016/j.eururo.2016.07.032 PMC5570461

[6] Shore ND, de Almeida Luz M, De Giorgi U, Gleave M, Gotto GT, Haas GP, et al. LBA02-09 EMBARK: a phase 3 randomized study of enzalutamide or placebo plus leuprolide acetate and enzalutamide monotherapy in high-risk biochemically recurrent prostate cancer. J Urol. 2023;210:224–6. 10.1097/JU.0000000000003518. 10.1097/JU.0000000000003518 37119051

[7] Armstrong AJ, Szmulewitz RZ, Petrylak DP, Holzbeierlein J, Villers A, Azad A, et al. ARCHES: a randomized, phase III study of androgen deprivation therapy with enzalutamide or placebo in men with metastatic hormone-sensitive prostate cancer. J Clin Oncol. 2019;37:2974–86. 10.1200/JCO.19.00799. 31329516 10.1200/JCO.19.00799 PMC6839905

[8] Armstrong AJ, Azad AA, Iguchi T, Szmulewitz RZ, Petrylak DP, Holzbeierlein J, et al. Improved survival with enzalutamide in patients with metastatic hormone-sensitive prostate cancer. J Clin Oncol. 2022;40:1616–22. 10.1200/JCO.22.00193. 35420921 10.1200/JCO.22.00193 PMC9113211

[9] Azad AA, Armstrong AJ, Alcaraz A, Szmulewitz RZ, Petrylak DP, Holzbeierlein J, et al. Efficacy of enzalutamide in subgroups of men with metastatic hormone-sensitive prostate cancer based on prior therapy, disease volume, and risk. Prostate Cancer Prostatic Dis. 2022;25:274–82. 10.1038/s41391-021-00436-y. 34420037 10.1038/s41391-021-00436-y

[10] Armstrong AJ, Shore ND, Szmulewitz RZ, Petrylak DP, Holzbeierlein J, Villers A, et al. Efficacy of enzalutamide plus androgen deprivation therapy in metastatic hormone-sensitive prostate cancer by pattern of metastatic spread: ARCHES post hoc analyses. J Urol. 2021;205:1361–71. 10.1097/JU.0000000000001568. 33356529 10.1097/JU.0000000000001568

[11] Stenzl A, Dunshee C, De Giorgi U, Alekseev B, Iguchi T, Szmulewitz RZ, et al. Effect of enzalutamide plus androgen deprivation therapy on health-related quality of life in patients with metastatic hormone-sensitive prostate cancer: an analysis of the ARCHES randomised, placebo-controlled, phase 3 study. Eur Urol. 2020;78:603–14. 10.1016/j.eururo.2020.03.019. 32336645 10.1016/j.eururo.2020.03.019

[12] Fizazi K, Foulon S, Carles J, Roubaud G, McDermott R, Fléchon A, et al. Abiraterone plus prednisone added to androgen deprivation therapy and docetaxel in de novo metastatic castration-sensitive prostate cancer (PEACE-1): a multicentre, open-label, randomised, phase 3 study with a 2 × 2 factorial design. Lancet. 2022;399:1695–707. 10.1016/S0140-6736(22)00367-1. 35405085 10.1016/S0140-6736(22)00367-1

[13] Smith MR, Hussain M, Saad F, Fizazi K, Sternberg CN, Crawford ED, et al. Darolutamide and survival in metastatic, hormone-sensitive prostate cancer. N Engl J Med. 2022;386:1132–42. 10.1056/NEJMoa2119115. 35179323 10.1056/NEJMoa2119115 PMC9844551

[14] Chowdhury S, Bjartell A, Agarwal N, Chung BH, Given RW, Pereira de Santana Gomes AJ, et al. Deep, rapid, and durable prostate-specific antigen decline with apalutamide plus androgen deprivation therapy is associated with longer survival and improved clinical outcomes in TITAN patients with metastatic castration-sensitive prostate cancer. Ann Oncol. 2023;34:477–85. 10.1016/j.annonc.2023.02.009. 36858151 10.1016/j.annonc.2023.02.009

[15] Sweeney CJ, Chen YH, Carducci M, Liu G, Jarrard DF, Eisenberger M, et al. Chemohormonal therapy in metastatic hormone-sensitive prostate cancer. N Engl J Med. 2015;373:737–46. 10.1056/NEJMoa1503747. 26244877 10.1056/NEJMoa1503747 PMC4562797

[16] Assayag J, Kim C, Chu H, Webster J. The prognostic value of Eastern Cooperative Oncology Group performance status on overall survival among patients with metastatic prostate cancer: a systematic review and meta-analysis. Front Oncol. 2023;13:1194718. 10.3389/fonc.2023.1194718. 38162494 10.3389/fonc.2023.1194718 PMC10757350

[17] Wu LW, Schwartz L, Bates SE. Regarding RECIST in retrospective studies. Oncologist. 2025;30:oyaf182. 10.1093/oncolo/oyaf182. 40578844 10.1093/oncolo/oyaf182 PMC12238941

[18] Heesterman BL, van der Poel HG, Schoots IG, Mehra N, Aben KKH. Prognostic importance of concomitant non-regional lymph node and bone metastases in men with newly diagnosed metastatic prostate cancer. BJU Int. 2022;130:217–25. 10.1111/bju.15632. 34741789 10.1111/bju.15632

[19] Stenzl A, Shore ND, Villers A, Iguchi T, Gomez-Veiga F, Alcaraz A, et al. Clinical outcomes of patients with metastatic Hormone-Sensitive Prostate Cancer (mHSPC) with Prostate-Specific Antigen (PSA) decline to undetectable levels on enzalutamide (ENZA): Post hoc analysis of ARCHES. Eur Urol. 2022;81:S776–7.

[20] Santoni M, Büttner T, Rescigno P, Fiala O, Cavasin N, Basso U, et al. Apalutamide in metastatic castration-sensitive prostate cancer: results from the multicenter real-world ARON-3 study. Eur Urol Oncol. 2025;8:444–51. 10.1016/j.euo.2024.11.005 39613567

[21] Saad F, Hussain MHA, Tombal B, Fizazi K, Sternberg CN, Crawford ED, et al. Deep and durable prostate-specific antigen response to darolutamide with androgen deprivation therapy and docetaxel, and association with clinical outcomes for patients with high- or low-volume metastatic hormone-sensitive prostate cancer: analyses of the randomized phase 3 ARASENS study. Eur Urol. 2024;86:329–39. 10.1016/j.eururo.2024.03.036. 38644146 10.1016/j.eururo.2024.03.036

[22] Matsubara N, Chi KN, Özgüroğlu M, Rodriguez-Antolin A, Feyerabend S, Fein L, et al. Correlation of prostate-specific antigen kinetics with overall survival and radiological progression-free survival in metastatic castration-sensitive prostate cancer treated with abiraterone acetate plus prednisone or placebos added to androgen deprivation therapy: post hoc analysis of phase 3 LATITUDE study. Eur Urol. 2020;77:494–500. 10.1016/j.eururo.2019.11.021. 31843335 10.1016/j.eururo.2019.11.021

[23] Maughan BL, Liu Y, Mundle S, Wang X, Nematian-Samani M, Karsh LI. Survival outcomes of apalutamide as a starting treatment: impact in real-world patients with metastatic hormone sensitive prostate cancer (OASIS). Prostate Cancer Prostatic Dis. 2025;28:865–73. 10.1038/s41391-024-00929-6 PMC12643925 39702472

