# Supplementary material for: Enzalutamide in metastatic hormone-sensitive prostate cancer: results from the international, multicentre, real-world ARON-3 study
Source: Prostate Cancer Prostatic Dis. 2026 Jan 8;29(3):588–94. doi: 10.1038/s41391-025-01067-3 (PMC13472872; doi:10.1038/s41391-025-01067-3)
Supplement: Supplementary file 1 [file 41391_2025_1067_MOESM1_ESM.docx]

**Supplementary Materials**

**Table S1.** Rate of Severe Adverse Events (SAEs) by age.

| **Toxicities** | **Overall population**  **%** | **Age <70y**  **%** | **Age ≥70y**  **%** |
| --- | --- | --- | --- |
| **Severe adverse events (overall)** | 9 | 8 | 10 |
| **G3-G4 fatigue** | 8 | 6 | 10 |
| **G3-G4 hypertension** | 2 | 2 | 2 |
| **G3-G4 rash** | <1 | <1 | <1 |
| **G3-G4 fractures** | <1 | <1 | <1 |
| **G3-G4 anemia** | <1 | <1 | <1 |
| **G3-G4 neutropenia** | <1 | <1 | <1 |

**Figure S1.** Selection process from the ARON-3 dataset.

**Figure S2.** Time on Treatment with enzalutamide in mHSPC patients, stratified by clinical characteristics.

**Figure S3.** Overall Survival with enzalutamide in mHSPC patients, stratified by type of visceral metastases.
